# Reference Genes for Addressing Gene Expression of Bladder Cancer Cell Models under Hypoxia: A Step Towards Transcriptomic Studies

**DOI:** 10.1371/journal.pone.0166120

**Published:** 2016-11-11

**Authors:** Luís Lima, Cristiana Gaiteiro, Andreia Peixoto, Janine Soares, Manuel Neves, Lúcio Lara Santos, José Alexandre Ferreira

**Affiliations:** 1 Experimental Pathology and Therapeutics Group – Research Center, Portuguese Institute of Oncology of Porto (IPO-Porto), 4200-072, Porto, Portugal; 2 i3S - Instituto de Investigação e Inovação em Saúde, Universidade do Porto, Portugal; 3 Institute of Molecular Pathology and Immunology of the University of Porto (IPATIMUP), 4200-135 Porto, Portugal; 4 Institute of Biomedical Sciences Abel Salazar (ICBAS), University of Porto, 4050-313 Porto, Portugal; 5 Health School of University of Fernando Pessoa, 4249-004, Porto, Portugal; 6 Department of Surgical Oncology, Portuguese Institute of Oncology of Porto (IPO-Porto), 4200-072, Porto, Portugal; Northwestern University Feinberg School of Medicine, UNITED STATES

## Abstract

Highly aggressive, rapidly growing tumors contain significant areas of hypoxia or anoxia as a consequence of inadequate and/or irregular blood supply. During oxygen deprivation, tumor cells withstand a panoply of adaptive responses, including a shift towards anaerobic metabolism and the reprogramming of the transcriptome. One of the major mediators of the transcriptional hypoxic response is the hypoxia-inducible factor 1 (HIF-1), whose stabilization under hypoxia acts as an oncogenic stimulus contributing to chemotherapy resistance, invasion and metastasis. Gene expression analysis by qRT-PCR is a powerful tool for cancer cells phenotypic characterization. Nevertheless, as cells undergo a severe transcriptome remodeling.in response to oxygen deficit, the precise identification of reference genes poses a significant challenge for hypoxic studies. Herein, we aim to establish the best reference genes for studying the effects of hypoxia on bladder cancer cells. Accordingly, three bladder cancer cell lines (T24, 5637, and HT1376) representative of two distinct carcinogenesis pathways to invasive cancer (FGFR3/CCND1 and E2F3/RB1) were used. Additionally, we have explored the most suitable control gene when addressing the influence of Deferoxamine Mesilate salt (DFX), an iron chelator often used to avoid the proteasomal degradation of HIF-1α, acting as an hypoxia-mimetic agent. Using bioinformatics tools (GeNorm and NormFinder), we have elected *B2M* and *HPRT* as the most stable genes for all cell lines and experimental conditions out of a panel of seven putative candidates (*HPRT*, *ACTB*, *18S*, *GAPDH*, *TBP*, *B2M*, and *SDHA*). These observations set the molecular basis for future studies addressing the effect of hypoxia and particularly HIF-1α in bladder cancer cells.

## Introduction

Hypoxia (decreased oxygen partial pressure from <0.1 mmHg (anoxia) to 15 mmHg) is a key microenviromental factor underlying malignant transformation, tumor heterogeneity, disease progression, immune escape, and therapy resistance [1–4]. In solid tumours, the uncontrolled proliferation of cancer cells combined with the structural abnormalities of the tumor vascular network results in the delivery of suboptimal concentrations of oxygen and other nutrients to cancer cells, creating hypoxic microregions [5, 6]. As a survival strategy, hypoxic cancer cells activate major adaptive pathways, and undergo a significant reprogramming of the cell transcriptional activity towards more aggressive phenotypes [7]. The key regulators of the transcriptional hypoxic response are the hypoxia-inducible factors (HIFs), which consist in one alpha subunit, whose expression is oxygen-dependent, and one constitutive beta subunit [6, 8, 9]. HIF-1α is constantly synthesized in the cytosol and rapidly degraded under normoxia; however, it becomes stabilized under low oxygen pressure. After nuclear translocation, HIF-1α directs the expression of multiple genes, promoting the overexpression of angiogenic and anti-apoptotic genes, as well as growth factors, glycolysis, and protein synthesis suppression [2, 10–13]. Moreover, hypoxic stress enhances motility and the invasive capacity of tumor cells via the acquisition of mesenchymal traits [14, 15]. Furthermore, it has been described to favor the establishment of cancer stem cell phenotypes, responsible by recapitulating tumor heterogeneity after treatment [16]. As such, the elimination of hypoxic cells, often responsible by disease relapse and progression, remains a challenging topic in cancer therapeutics.

The transcriptomic analysis of hypoxic cells is of primary importance to understand the roles played by genes in the overall cellular responses and, for ultimately setting the molecular basis for targeted therapeutics. The quantitative reverse-transcription real-time polymerase chain reaction (qRT-PCR) is the gold standard technique to access gene expression through the quantification of mRNA levels [17]. Furthermore, it is frequently used to validate the results from microarray experiments [18]. Even though considered highly sensitive, reproducible and with wide dynamic range, qRT-PCR experiments require the normalization of the results to a reference gene [19, 20]. This internal control is subjected to the same preparation steps as the genes of interest, allowing to refine non-biological variations resulting from sample manipulation [21]. Ideally, it should be uniformly expressed in all experimental conditions of a given systems, and its expression should be similar to the target genes [18]. In particular, exposure to hypoxia poses a hurdle for transcriptomic studies, since it has been shown to modulate the transcription of classical and widely explored reference genes, namely glyceraldehyde-3-phosphate dehydrogenase (*GAPDH)*, β-actin (*ACTB)*, and β-tubulin *(TUBB)* in several cancer cell lines [22–26]. Still, significant variations have been observed throughout the literature depending on the used model, and many hypoxia-related transcriptomic studies overlooked the careful identification of the most suited reference genes for their particular experimental context.

Recent evidences suggest that hypoxia plays a key role in bladder cancer chemoresistance, invasion and dissemination, which warrants future comprehensive studies towards novel biomarkers and innovative therapeutics [27–30]. Based on these observations, we have devoted to the identification of the most stable reference genes out of a panel of seven candidates, namely Hypoxanthine phosphoribosyltransferase-1 *(HPRT)*, *ACTB*, 18 ribosomal RNA (*18S)*, *GAPDH*, TATA-binding protein (*TBP)*, Beta-2 microglobulin (*B2M)*, and Succinate dehydrogenase complex flavoprotein subunit A (*SDHA*), to address the effect of hypoxia in the context of bladder cancer. We have selected three bladder cancer cell lines (T24, 5637, and HT1376), representative of two distinct molecular pathways to invasive cancer (FGFR3/CCND1and E2F3/RB1), and widely explored by us in the establishment of novel therapeutic schemes. Additionally, we have also explored the most suitable reference gene in the presence of the hypoxia-mimetic Deferoxamine Mesilate salt (DFX), which has been widely applied to disclose HIF-1α-mediated events by avoiding the proteasomal degradation of the transcription factor under normoxia. Altogether we envisage to create the necessary rationale for high throughput transcriptomic and (glyco)proteomic studies towards the identification of novel bladder cancer biomarkers associated with hypoxia.

## Material and Methods

### Cell lines and culture conditions

The T24 (grade III), 5637 (grade II) and HT1376 (grade III) bladder cancer cell lines used in this work were acquired from DSMZ (Düsseldorf, Germany) and recently characterized from the genetic standpoint by our group [31]. Accordingly, the T24 cell line is representative of the FGFR3/CCND1 pathway of invasion, presenting a mutated HRAS and overexpression of CCND1. The 5637 and HT1376 cell lines correspond to the E2F3/RB1 invasion pathway with loss of one copy of RB1 and mutation of the remaining copy. Additionally, HT1376 cells exhibit deletion of the Phosphatase and tensin homolog (*PTEN)* gene and no alteration of Phosphatidylinositol 3-kinase catalytic subunit alpha (*PIK3CA)*, which in combination with the inactivation of p53, translates into a more invasive and metastatic potential. In contrast, the 5637 cell line presents *PIK3CA* gene deletion and no *PTEN* alterations, which translates into a less-invasive phenotype.

The cells were cultured in RPMI 1640+GlutaMAXTM-I medium (Gibco, Life Technologies) supplemented with 10% heat-inactivated FBS (Gibco, Life Technologies) and 1% penicillin-streptomycin (10,000 Units/mL P; 10,000 μg/mL S; Gibco, Life Technologies). Cell lines were cultured as a monolayer at 37°C in a 5% CO2 humidified atmosphere (normoxia), and were routinely subcultured after trypsinization. The cells were also grown under hypoxic atmosphere for 24h (T24 and 5637) or 6h (HT1376) at 37°C with 5% CO2, 99.9% N2 and 0.1% O2 in a BINDER C-150 incubator (BINDER GmbH). Additionally, cells were grown under normoxia in the presence of 500 μM Deferoxamine Mesilate CRS (DFX, Sigma-Aldrich), a hypoxia-mimetic agent that promotes HIF-1α stabilization [32]. Cell viability was determined using the Trypan Blue Exclusion Test of Cell Viability. Of note, cells maintained nearly 100% viability for all experimental conditions.

### Expression of hypoxia makers

Growth under hypoxia was confirmed by evaluation of hypoxia marker HIF-1α by western blot using the rabbit anti-human HIF-1α clone [16H4L13] (1:250 in PBS; Invitrogen) as primary antibody. The Abcam’s fluorometric L-Lactate assay kit (Abcam) was used to determine the concentration of L-Lactate in culture media as described by the supplier. A significant increase in lactate concentration was considered as a surrogate marker of anaerobic metabolism.

### RNA isolation and cDNA conversion

Total RNA was isolated from T24, 5637 and HT1376 cells, grown under normoxia, hypoxia and DFX exposure, using TriPure isolation Reagent (Roche Diagnostics GmbH, Mannheim, Germany), according to the manufacturer’s instructions. The quality and quantity of the extracted RNA was estimated using a Nanodrop (ND1000, Nano Drop Technologies Inc. Wilmington, DE, USA). Before cDNA synthesis, the integrity of RNA samples was confirmed by electrophoresis on 1% agarose gels. RNA (2μg) was reverse transcribed with random primers, using the “High Capacity cDNA Reverse Transcription Kit” (Applied Biosystems, Foster City, CA).

### mRNA expression analysis

Candidate reference genes were selected according to the following criteria: (i) high frequency of use (*ACTB* and *GAPDH*); (ii) used for normalization in other hypoxia studies, cancer types and models (*HPTR*, *TBP*, *B2M*, *SDHA*, *18S*). *CA9* was also evaluated as a control since it is a transcript that is regulated by hypoxia/DFX. Real-time PCR amplification of cDNA samples was performed in a ABIPrism 7500™ Real-Time PCR System (Applied Biosystems) using a TaqMan^®^ Gene Expression Master Mix, primers and probes provided by Applied Biosystems. The S1 Table shows the manufacturer references for each evaluated genes. The thermal cycling conditions comprised an initial denaturation step at 95°C for 30 s, 40 cycles at 95°C for 5 s, a 34 s period at 65°C, and a final dissociation stage at 95°C for 15 s, 60°C for 1 min, and 95°C for 15 s [33]. All experiments were performed in duplicates. For each experiment, two conversion replicates were done and for every conversion replicate three amplification replicates were performed, resulting a 12 replicates total for each experimental condition.

### Analysis of gene expression stability by RT-qPCR

The stability of the reference genes expression was assessed using two different statistical programs: geNorm [34] and NormFinder [35]. The geNorm software analyses the stability of reference genes transcripts taking into account the expression stability value (M^geNorm^) [36]. The this stability value is calculated for each gene of a panel of candidate reference genes based on pairwise variation analysis. Moreover, and according to Vandesompele *et al*., *l*ower values of M^geNorm^ correspond to higher gene expression stability [34]. Furthermore, geNorm is also capable to determine the ideal number of reference genes needed for accurate normalization [34]. Similarly, NormFinder, is a mathematical algorithm used to identify the best normalization gene amongst a set of reference candidates according to their expression stability (M ^NormFinder^), analyzing both intra- and inter-group variation [22]. Like GeNorm, lower average M ^NormFinder^ values indicate more stable genes expression [35].

## Results

**Fig 1:**
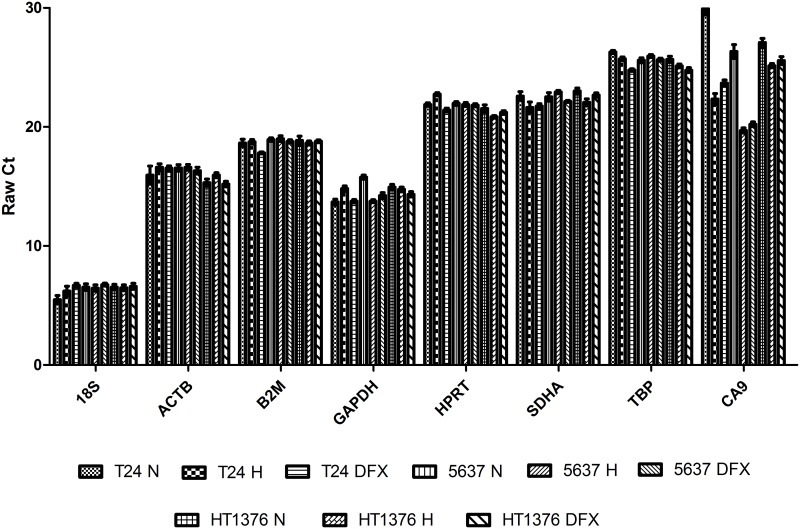
Expression of the evaluated genes. Raw mean Ct values of the reference genes (*HPRT*, *ACTB*, *18S*, *GAPDH*, *TBP*, *B2M*, *SDHA)* and *CA9* for T24, 5637 and HT1376 cell lines under different experimental conditions (Normoxia (N), Hypoxia (H), and DFX). Results are presented as mean + SD. Twelve replicates were used.

The present work was devoted to the identification of references genes for accessing gene transcription alterations associated with hypoxia in bladder cancer. Three of the most studied bladder cancer cell lines (T24, 5637, and HT1376), reflecting different molecular pathways for cell invasion (FGFR3/CCND1and E2F3/RB1), were chosen envisaging the identification of a ubiquitous reference gene. Accordingly, seven of the most commonly used reference genes (*ACTB*, *GAPDH*, *HPRT*, *B2M*, *SDHA*, *TBP*, and *18S)* were amplified in the three bladder cancer cell lines exposed to normoxia and hypoxia. In addition, experiments were conducted in the presence of DFX to disclose HIF-1α mediated events. All cell lines showed increased levels of HIF-1α and lactate under hypoxia and DFX in relation to normoxia, denoting a shift towards anaerobic metabolism, thereby confirming a cellular response to hypoxic stress (S1 Fig). The variation between the maximum and minimum Ct (cycle threshold) values for each tested reference gene was less than 3 cycles for all experimental conditions and cell lines (Fig 1). Particularly, *18S* was the most expressed reference gene regardless the cell line and experimental condition, with a mean Ct value of approximately 6.5. *GAPDH* was the second most transcribed gene with a mean Ct of 14.5, and *TBP* was the least expressed gene with Ct values of 25.5. Moreover, our control gene *CA9* presented a mean expression level of 24.5. As it can be seen the *CA9* Ct values in hypoxia and DFX conditions are lower than the normoxia in all cell lines, showing that this HIF-regulated gene is robustly upregulated in these conditions.

**Fig 2:**
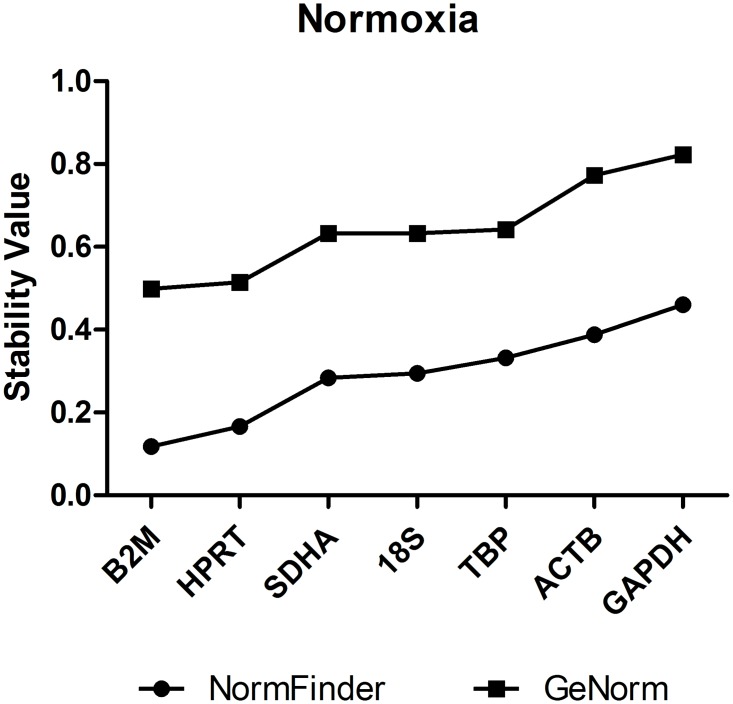
Ranked expression stability of the reference genes (geNorm and NormFinder analysis) under normoxic conditions for all cell lines. Under normoxia, *B2M* and *HPRT* presented the lower stability values, meaning they are the most stable reference genes in this condition. On the other hand, *ACTB* and *GAPDH* presented the higher stability values, being the least stable genes. Of note, both analytical tools provided similar outcomes.

**Fig 3:**
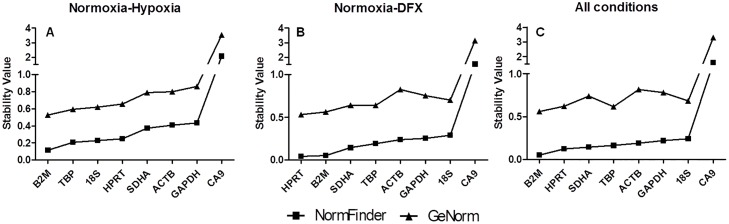
geNorm and NormFinder analysis of the stability values of reference genes and *CA9*. Samples were exposed to (A) normoxia and hypoxia, (B) normoxia and DFX, and (C) all experimental conditions (normoxia, hypoxia and DFX). *B2M* and *HPRT* are constitutively top ranked while *ACTB* and *GAPDH* are low ranked regardless of the experimental condition.

To reliably identify the most stably expressed genes across all cell lines and experimental conditions, both geNorm and NormFinder statistical programs were used. Of note, the two software originated highly comparable results (S2 Fig). geNorm and NormFinder retrieved *B2M* and *HPRT* as the most stable reference genes under normoxic conditions for all cell lines, followed by *SDHA*, *18S* and *TBP*. *ACTB* and *GAPDH* were found to be the most unstable genes among the studied candidates (Fig 2). Moreover, when comparing normoxic and hypoxic conditions, the most stable candidate gene for each cell line was *B2M*, followed by *TBP*, *18S* and *HPRT*, which presented similar stability values. This results suggest that all of the above reference genes could be suitable controls in combination with *B2M* (Fig 3). Again, *GAPDH* and *ACTB* were shown to be the most unstable genes under hypoxic conditions. Reinforcing the consistent stability of a good reference gene, when comparing normoxia with DFX exposure conditions, *B2M* and *HPRT* were again shown to be the most stable genes, while *ACTB*, *GAPDH*, and *18S* were the less stable (Fig 3B).

**Fig 4:**
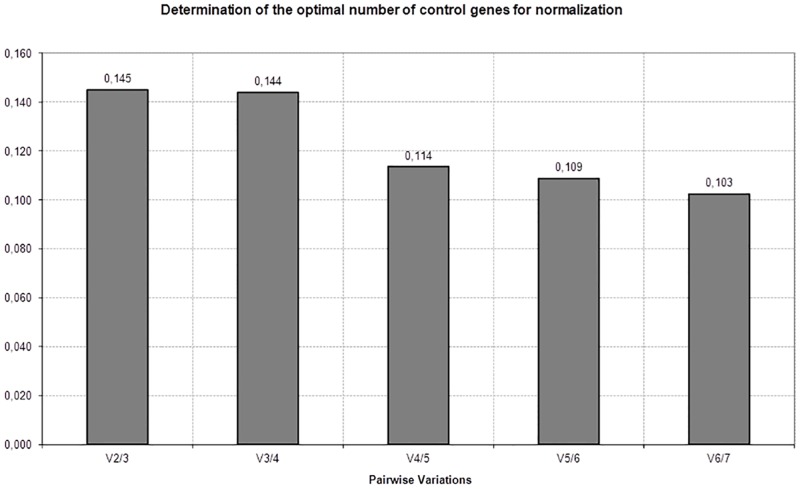
Determination of the optimal number of control genes for accurate normalization using geNorm software. Each bar V_*n/n+1*_ represents the variation between the means of the *n* most stable genes versus the group of *n+1* most stable genes. For example, column 1 represents the variation between the mean of the two most stable genes, *B2M* and *HPRT*, versus the three most stable genes *B2M*, *HPRT* and *SDHA*. Because the 0.15 threshold is not exceeded at any point, the use of two reference genes would be sufficient under these conditions.

**Fig 5:**
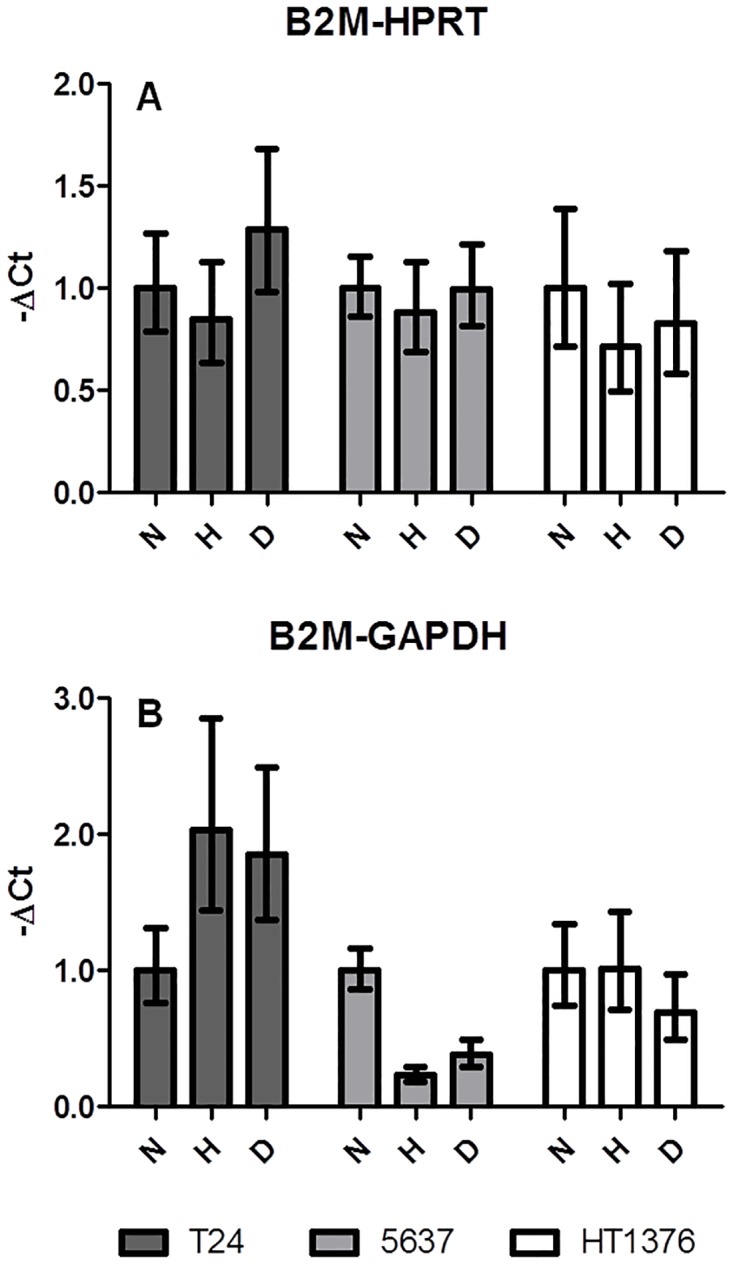
Examples of gene expression calculations. Relative mRNA expression calculated by normalization of a stably expressed gene (*B2M*) to another stable gene (*HPRT*, A) or to an unstable gene (*GAPDH*, B) under different experimental conditions: normoxia (N), hypoxia (H), DFX (D).

Finally, all experimental conditions were compared in order to identify a common suitable reference gene for further comprehensive studies (Fig 3C). This approach highlighted *B2M* and *HPRT* as the most stable genes for all cell lines among all control genes tested. Nevertheless, geNorm analysis showed a stability value for *TBP* similar to *HPRT*, while NormFinder analyses ranked *TBP* only as the fourth most stable. Again, *GAPDH*, *18S* and *ACTB* were ranked the least stable genes of the evaluated panel. We have also analyzed the stability value of *CA9*, a gene which expression is known to be affected under hypoxia. Has expected the stability values for *CA9* were much more higher, reinforcing the idea that this cell are really in hypoxia (Fig 3). Since the proper normalization of expression data determines the accuracy of RT-qPCR quantification, we have determined the optimal number of reference genes for data normalization through a pairwise variation (V_n/n+1_) analysis using GeNorm, (Fig 4). As suggested by Vandesompele, *et al*, we have adopted a cut-off of V_n/n+1_<0.15 as an appropriate selection criterion for estimating the optimal number of reference genes [34]. Of note, this cut-off value has been widely used as a criterion for selection of reference genes [37–41]. As shown in Fig 5, the value V2/3 is 0.145, indicating that the inclusion of a third reference gene would not improve the accuracy obtained with only two reference genes.

To demonstrate that the selection of an inappropriate reference gene for normalization might lead to erroneous data interpretations we have normalized *B2M* using *HPRT*. Accordingly, *B2M* expression was demonstrated to be reasonably stable in all cell lines and experimental conditions (Fig 5A), highlighting the high stability of both genes. On the other hand, when *B2M* was normalized to *GAPDH*, one of the less stable genes of the tested panel, significant variations could be observed. Namely, *B2M* showed increased expression in T24 cells exposed to hypoxia and DFX, and lower expression in 5637 cells exposed to the same conditions. Moreover, DFX-exposed HT1376 cells presented a downregulation of *B2M* (Fig 5B). These results highlighted that an appropriate normalization across samples determines the magnitude of relative expression levels of the genes tested.

## Discussion

Significant amount of evidences support the notion that hypoxia enhances the malignant nature of bladder cancer cells by promoting tumor cell migration, invasion, chemoresistance, metastasis and immune modulation [28, 30, 42–44]. Furthermore, many of the transcriptome remodeling events underlying these transformations are mediated by HIF-1α [45]. Interestingly, many studies addressing gene expression in hypoxic cancer cells have disregarded the adaptations of classical and widely explored reference genes to microenvironmental challenges, which lead to the flawed election of targets for downstream analysis [46]. A common example includes *GAPDH*, which is significantly upregulated under low oxygen tension in many models, making it an unsuitable reference gene for hypoxic studies [23, 46, 47]. Accordingly, we have also found that *GAPDH* was among the least stable genes in the studied bladder cancer cells under hypoxia. Still, some reports support *GAPDH* as an excellent reference gene in hypoxic hepatocellular, colonic and lung carcinoma models [24]. In addition, hypoxic cells frequently undergo epithelial-to-mesenchymal transition (EMT), a plastic process in which fully differentiated epithelial cells are converted into poorly differentiated, migratory and invasive mesenchymal cells [48]. This comprehends a profound remodeling of the cytoskeleton, which includes a downregulation of *ACTB*, also a widely used reference gene [49, 50]. Likewise, we have observed that *ACTB* gene was unstable in bladder cancer cell lines exposed to hypoxia. Nevertheless, contradictory evidences have emerged regarding the election of *ACTB* as a reference gene for hypoxic studies. For instance, *ACTB* has been considered a stable control gene in breast (MCF-7 cell line) [46] and prostate cancer (LNCaP, 22Rv1, PC3, and DU14) cell lines under hypoxia [25]. These observations highlight that gene stability under hypoxia is highly dependent on the origin of cells as well as the necessity to undergo careful transcriptome evaluation before more in depth molecular studies.

As previously mentioned, RT-qPCR data quantification still remains challenging due to the random selection and number of reference genes used for data normalization in most studies. Moreover, many reports still rely just on a single reference gene for data normalization, despite the existence of more robust statistical methods for the evaluation of several controls. Herein, we have employed geNorm, first developed by Vandesompele, *et al* [34], and NormFinder statistical programs to access the stability of candidate reference genes. Both retrieved highly comparable results, especially in terms of gene stability ranking [51]. Accordingly, we have identified *B2M* and *HPRT* as the most stable reference genes to address the impact of oxygen shortage in hypoxic bladder cancer cells, irrespectively of their molecular nature. This was also verified in cells exposed to DFX [32], suggesting these genes are not regulated by HIF-1α. Moreover, both analytical programs have elected *GAPDH* and *ACTB* as the most unstable genes for all conditions.

Our study is in agreement with previous studies describing the stability of *B2M* in hypoxic cultured human chondrocytes [52]. Contrastingly, it has been reported that *B2M* expression is significantly altered in hypoxic prostate cancer cells out of a panel of 16 reference genes for qRT-PCR [25]. Similarly to bladder cancer cells, hypoxic prostate cancer and neural stem cells have also demonstrated a stable *HPRT* expression using geNorm and NormFinder [53, 54]. In contrast, Tan *et al*., using a model of endogenous cardiac stem cells, suggested that *HPRT* transcription is not stable under hypoxic conditions [55]. In summary, these findings reinforce the notion that the stable expression of a reference gene is context-dependent and may significantly vary between models. Which poses a limitation of this work, the results stated herein only apply to the three bladder cancer cell lines used in our experiments. Even though these cell lines are the most studied regarding bladder cancer *in vitro* research, only represents a subset of all bladder cancer cell lines available.

The identification of an optimal number of reference genes is also important for accurate normalization of RT-qPCR data, especially when differences in expression levels are subtle. According to geNorm software, two is the minimal number of reference genes, namely *B2M* and *HRPT*, for obtaining an accurate normalization under the hypoxic conditions in the studied cell bladder cancer lines.

In summary, we have highlighted possible problems when translating results from different models in the context of hypoxia, and reinforced the need for careful evaluation of reference genes prior to more in depth molecular studies. To the best of our knowledge this study is the first to analyze and evaluate the stability of a set of reference genes in the hypoxic context for these three bladder cancer cell lines. This information is important for other researchers that will need to evaluate mRNA expression on these cell lines and in this context, using the most widely used tool for gene expression analysis. Moreover, we have set the molecular basis to comprehensively address the transcriptional programs and molecular nature of bladder cancer cells under hypoxia, as well as the role of HIF-1α, towards novel biomarkers and therapeutic strategies.

## Supporting Information

S1 Fig Evaluation of HIF-1α, CAIX and lactate levels as controls of hypoxia induction. Western Blot analysis of HIF-1α and CAIX proteins, 24h (T24 and 5637 cell lines) and 6h (HT1376 cell line) after treatment **(A)**. B2M was used as loading control. The western blot samples appear in the following order: T24, 5637 and HT1376 Normoxia (N); T24, 5637 and HT1376 Hypoxia (H); T24, 5637 and HT1376 DFX treatment (D). Molecular weight markers (MWM) are expressed in kDa. Bladder cancer cell lines overexpressed HIF-1α **(B)** and CAIX **(C)** hypoxia biomarkers when exposed to hypoxia. Concomitantly, the metabolic shift from aerobic to anaerobic metabolism, a critical event underlying hypoxia, was also confirmed by increased lactate levels in hypoxia treated cell culture mediums **(D)**. The stabilization of HIF-1α with DFX resulted in similar behaviours suggesting that this transcription factor might regulate this events.
(TIF)

S2 Fig Average expression stability values of the 7 candidate reference genes for all cell lines analysed using geNorm and NormFinder approaches. The individual ranks provided by both softwares for each cell line under all studied conditions are summarized: (A-C) T24 cell line comparing (A) normoxia and hypoxia, (B) normoxia and Dfx, (C) and three studied conditions. (D-F) 5637 cell line comparing (D) normoxia and hypoxia, (E) normoxia and Dfx, (F) and three studied conditions. (G-I) HT1376 cell line comparing (G) normoxia and hypoxia, (H) normoxia and Dfx, (I) and all studied conditions.
(TIF)

S1 Table TaqMan-based gene expression assays references used to mRNA expression analysis for the 7 candidate reference genes. (DOCX)
