# Supplementary figures and images for: Reference Genes for Addressing Gene Expression of Bladder Cancer Cell Models under Hypoxia: A Step Towards Transcriptomic Studies
Source: PLoS One. 2016 Nov 11;11(11):e0166120. doi: 10.1371/journal.pone.0166120 (PMC5106008; doi:10.1371/journal.pone.0166120)

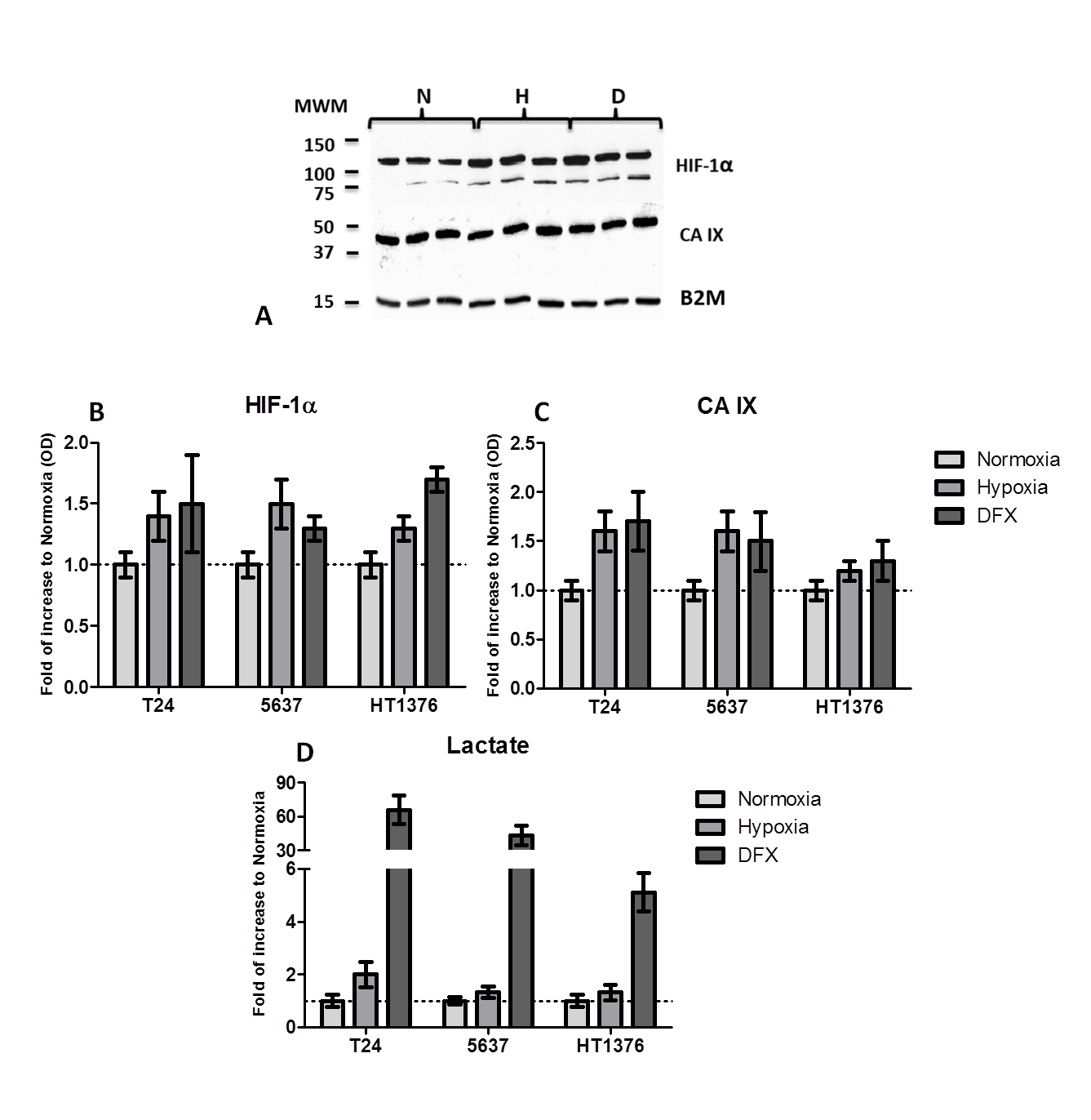

Supplement: S1 Fig — Western Blot analysis of HIF-1α and CAIX proteins, 24h (T24 and 5637 cell lines) and 6h (HT1376 cell line) after treatment (A). B2M was used as loading control. The western blot samples appear in the following order: T24, 5637 and HT1376 Normoxia (N); T24, 5637 and HT1376 Hypoxia (H); T24, 5637 and HT1376 DFX treatment (D). Molecular weight markers (MWM) are expressed in kDa. Bladder cancer cell lines overexpressed HIF-1α (B) and CAIX (C) hypoxia biomarkers when exposed to hypoxia. Concomitantly, the metabolic shift from aerobic to anaerobic metabolism, a critical event underlying hypoxia, was also confirmed by increased lactate levels in hypoxia treated cell culture mediums (D). The stabilization of HIF-1α with DFX resulted in similar behaviours suggesting that this transcription factor might regulate this events. (TIF) [file pone.0166120.s001.tif]

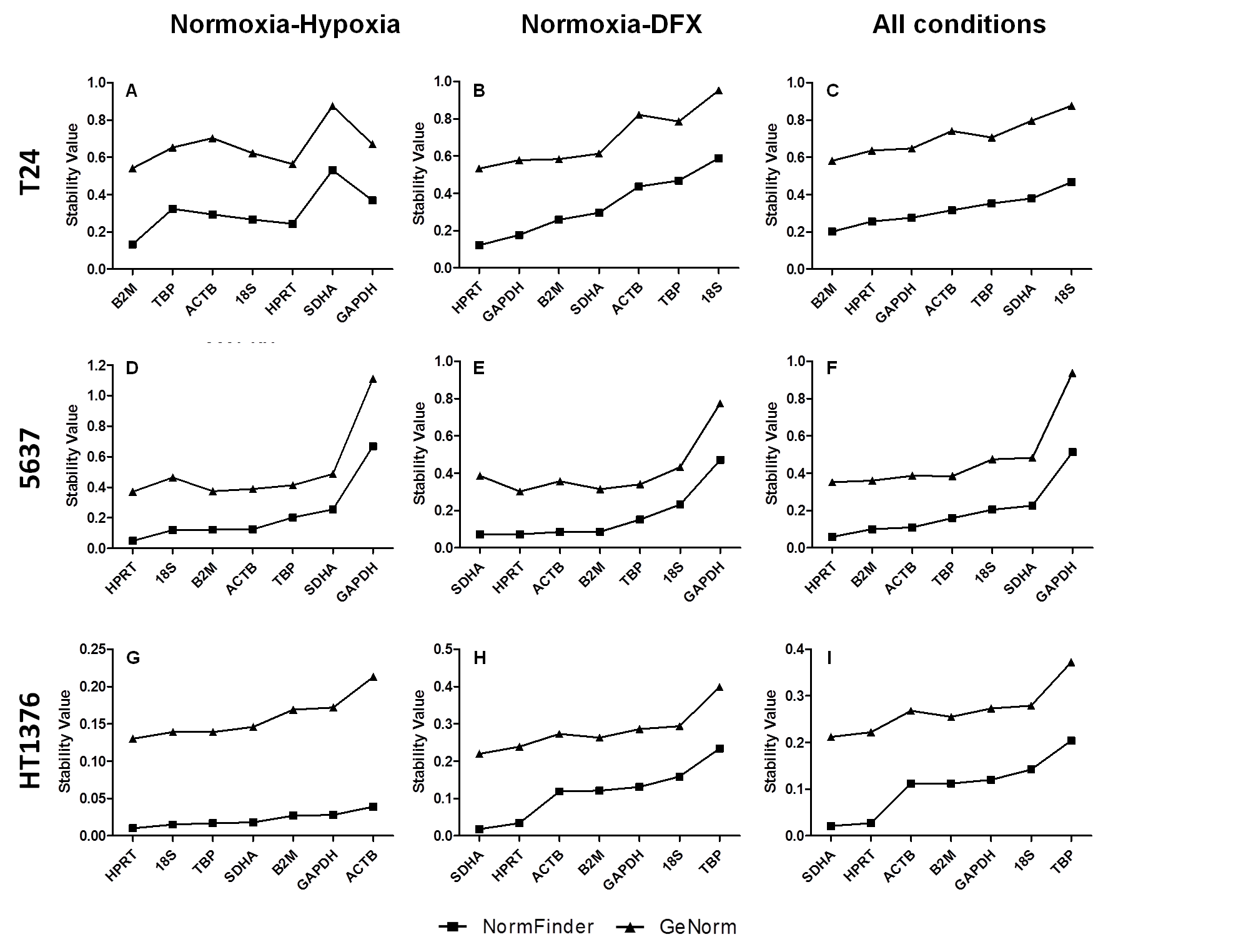

Supplement: S2 Fig — The individual ranks provided by both softwares for each cell line under all studied conditions are summarized: (A-C) T24 cell line comparing (A) normoxia and hypoxia, (B) normoxia and Dfx, (C) and three studied conditions. (D-F) 5637 cell line comparing (D) normoxia and hypoxia, (E) normoxia and Dfx, (F) and three studied conditions. (G-I) HT1376 cell line comparing (G) normoxia and hypoxia, (H) normoxia and Dfx, (I) and all studied conditions. (TIF) [file pone.0166120.s002.tif]
