# Supplementary material for: Reference Genes for Addressing Gene Expression of Bladder Cancer Cell Models under Hypoxia: A Step Towards Transcriptomic Studies
Source: PLoS One. 2016 Nov 11;11(11):e0166120. doi: 10.1371/journal.pone.0166120 (PMC5106008; doi:10.1371/journal.pone.0166120)
Supplement: S1 Table — (DOCX) [file pone.0166120.s003.docx]

Table S1 – TaqMan-based gene expression assays references used to mRNA expression analysis for the 7 candidate reference genes.

| **Reference Gene** | **Complete description** | **Assay Reference** |
| --- | --- | --- |
| *ACTB* | β-actin | Hs99999903_m1 |
| *GAPDH* | Glyceraldehyde-3-phosphate dehydrogenase | Hs03929097_g1 |
| *HPRT* | Hypoxanthine phosphoribosyltransferase-1 | Hs99999909_m1 |
| *TBP* | TATA-binding protein | Hs00427620_m1 |
| *B2M* | Beta-2 microglobulin | Hs00984230_m1 |
| *SDHA* | Succinate dehydrogenase complex flavoprotein subunit A | Hs00188166_m1 |
| *18S* | 18 ribosomal RNA | Hs99999901_s1 |
| *CA9* | Carbonic anhydrase 9 | Hs00154208_m1 |
